# Improvement of EMAT Butterfly Coil for Defect Detection in Aluminum Alloy Plate

**DOI:** 10.3390/ma18133207

**Published:** 2025-07-07

**Authors:** Dazhao Chi, Guangyu Sun, Haichun Liu

**Affiliations:** 1 State Key Laboratory of Precision Welding & Joining of Materials and Structures, Harbin Institute of Technology, Harbin 150001, China; 22s009142@stu.hit.edu.cn; 2 PipeChina Engineering Quality Supervision and Inspection Company, Beijing 100013, China; liuhc@pipechina.com.cn

**Keywords:** electromagnetic acoustic transducer, aluminum alloy, finite element simulation, orthogonal experiment, defect detection

## Abstract

For non-destructive testing (NDT) of defects in aluminum alloy plates, traditional ultrasonic contact methods face challenges from high temperatures and liquid couplant contamination. Using electromagnetic acoustic transducers (EMATs), a key issue is that longitudinal waves (L-waves) excited by the butterfly-coil EMATs interfere with the desired shear waves (S-waves) reflected by internal defects. To solve this problem, a simulation–experiment approach optimized the butterfly coil parameters. An FE model visualized the electromagnetic acoustic transducer (EMAT) acoustic field and predicted signals. Orthogonal simulations tested three main parameters: excitation frequency, wire diameter, and effective coil width. Tests on aluminum specimens with artificial defects used the optimized EMAT. Simulated and measured signals showed strong correlation, validating optimal parameters. The results confirmed suppressed L-wave interference and improved defect detection sensitivity, enabling detection of a 3 mm diameter flat-bottomed hole buried 37 mm deep.

## 1. Introduction

Aluminum alloy rolled plates are extensively employed in modern industries due to their exceptional strength-to-weight ratio, corrosion resistance, and formability [1,2]. However, the rolling process inherently introduces internal defects such as pores, inclusions, and delaminations, which pose significant threats to the mechanical integrity and operational safety of the end products [3,4,5]. Non-destructive testing (NDT) technologies play a critical role in maintaining quality control and detecting these defects without compromising material performance [6,7].

Conventional contact-based ultrasonic testing methods, including Time-of-Flight Diffraction (TOFD) and ultrasonic phased array (PAUT), have been widely adopted in aluminum alloy plate and weldment inspection [8]. Zheng et al. [9] proposed an image-processing method combining the least-squares method and volumetric rendering. By processing the defect information obtained from TOFD inspection, they reconstructed the three-dimensional shape of the hole defects in the aluminum plate specimens, providing an effective method for the three-dimensional visualization of transverse hole defects in plates. For instance, Cong et al. [10] developed a multi-scanning TOFD approach to inspect 165 mm thick electron beam-welded joints by combining double-sided inspections with variable transducer spacings and refraction angles. This configuration ensured full coverage of the weld volume through two scanning passes. Jin et al. [11] addressed the near-surface blind zone issue in thin aluminum plates (7.0 mm) by introducing half-skip mode conversion waves, reducing the blind zone by 38% and achieving defect location errors within 5%. In order to evaluate the defects located on the same surface and at one side of the piezoelectric ultrasonic transducer array, Wong et al. [12] proposed a simplified algorithm for efficiently detecting and mapping the growth of surface defects located on the same surface and at one side of the piezoelectric ultrasonic transducer array based on the detection mode of the twice-reflected ultrasonic bulk wave (TRBW). This algorithm can detect incremental surface defect growth starting from 2.80 mm in depth. PAUT techniques have also demonstrated advantages for aluminum alloy plate and weldment. Nanekar et al. [13] developed an integrated approach to sound beam focusing by combining phased-array technology with the synthetic aperture focusing technique (PA-SAFT). This approach uses fewer array elements than those required by the conventional phased array to achieve sound beam focusing. The effectiveness of this approach was demonstrated on aluminum blocks with artificial flaws and steel plate samples with embedded volumetric weld flaws such as slag and clustered porosities. Shang et al. [14] optimized acoustic field parameters through precise delay law control, enhancing focusing efficiency. Lukomski et al. [15] implemented wave migration algorithms for ultrasonic imaging in friction-stir welds, successfully detecting voids and incomplete penetrations. Liu et al. [16] improved PAUT imaging quality by refining delay accuracy.

The electromagnetic ultrasonic method is a new type of ultrasonic testing technology. Some of the main advantages of EMATs over contacted UT include the fact that no couplant and surface preparation is required during measurement; thus, the cost of surface preparation during measurement and irregularities that arise from the use of a couplant are eliminated [17,18,19]. Compared with the electromagnetic ultrasonic, laser ultrasonic sources are relatively expensive. Moreover, high-power laser beams damage the surface of the workpiece, creating surface ablation pits [20]. However, an EMAT, as a transducer, has some inherent deficiencies that affect and limit its operation on materials. These deficiencies are its low signal-to-noise ratio (SNR) when compared with the conventional piezoelectric transducer, the size of the transducer, and the undesirable effect of using a permanent magnet [21]. For the field of electromagnetic UT, its optimization methods mainly include FE analysis, structural design, and combination with other UT methods. In the field of finite element analysis of electromagnetic ultrasonic waves, optimizing the structure and relevant parameters of the transducer through FE analysis is the main research direction. Zhai et al. [22] carried out an analytical modeling of the spiral-coil electromagnetic ultrasonic transducer. They studied multiple issues such as the magnetic field, Lorentz force, and design principles. The calculation speed of this model is dozens of times faster than that of the finite element method. Wang et al. [23] conducted research on the acoustic field directivity of butterfly-coil EMATs, constructed an experimental system for measuring acoustic field directivity, and analyzed the acoustic field characteristics under different operating frequencies and different polarization directions relative to semicircular specimens. Huang et al. [24] optimized five factors of the point-focusing electromagnetic acoustic transducer (PF-EMAT) through orthogonal experimental simulation. These factors include line width, lift-off distance, excitation current amplitude, bandwidth coefficient, and the number of coil turns. As a result, the size of the focal line was reduced by 15%. In the field of structural design of electromagnetic ultrasonic transducers, optimizing the structures of permanent magnets and coils can often greatly improve the performance. In order to suppress the multi-modal and dispersive characteristics of Lamb waves, Fan et al. [25] proposed a dual-row linear Halbach (DHB) electromagnetic acoustic transducer (EMAT) based on the linear Halbach array, the traditional electromagnetic acoustic transducer (EMAT), and the periodic permanent magnet (PPM) electromagnetic acoustic transducer. This transducer can achieve a 27.36-fold enhancement of the S0 wave signal in the received signal. In order to overcome the drawback of the low energy-conversion efficiency of electromagnetic ultrasonic Rayleigh waves, Chen et al. [26] designed a dual-curved meander line coils electromagnetic acoustic transducer (DC-MLCs EMAT) that can generate unidirectional, focused Rayleigh waves. The simulation and experimental results show that compared with the existing single constant length meander line coil (SCL-MLC) electromagnetic acoustic transducer, the signal strength is increased by 164.36%. Zhang et al. [27] optimized an enhanced flexible electromagnetic ultrasonic transducer for pipeline inspection. By using an array magnetic core in the racetrack coil-type flexible electromagnetic magnet to replace the traditional bias permanent magnet, the ultrasonic energy conversion efficiency was improved. It could detect the cracks on the outer surface of an aluminum pipe with a depth of 0.5 mm and enhanced the crack echo by more than 80%. Jaime et al. [28] designed a coil structure composed of two orthogonal coils. They detected vertical crack defects at the bottom of the material through the amplitude difference of the signals received by the two coils. Martinho et al. [29] proposed a novel apodized diamond-shaped periodic permanent magnet electromagnetic acoustic transducer (PPM EMAT) in order to reduce the side-lobe level in the radiation pattern of the PPM EMAT. That is, the size of the magnets in the PPM array is changed to a shape similar to a diamond, and the width of the magnets gradually decreases as the distance of the magnets from the center of the transducer increases. Compared with the traditional PPM EMAT, the side-lobe suppression effect of this novel EMAT can reach up to approximately 8 decibels at most. Gautam et al. [30] proposed a novel design of a chevron electromagnetic acoustic transducer (EMAT) to excite orthogonally polarized shear–horizontal (SH) guided waves. With this design, SH waves of different frequencies can be simultaneously generated in orthogonal directions to detect crack defects in aluminum alloy plates. Jiang et al. [31] proposed using pulse compression technology and non-equidistant coil structures to improve the signal quality. The results show that the amplitude of the received signal increased by 2.3 to 2.6 times, and the signal-to-noise ratio increased by 7.1 to 10.1 dB. In the electromagnetic ultrasonic non-destructive testing, combining electromagnetic ultrasonic testing technology with other testing methods can also play a good role in optimizing the performance. Dixon et al. [32] developed a high-power phased-array electromagnetic ultrasonic transducer with racetrack coils as array elements. Compared with a single coil, the signal intensity increased by 3.5 times, and the SNR increased by 1.9 times. Shi et al. [33] aimed at the problem of the relatively low SNR of the ultrasonic echoes generated in metallic materials by the ultrasonic method using a laser-electromagnetic ultrasonic transducer (laser-EMAT). They proposed and analyzed the influence of the water film surface constraint on the ultrasonic detection echoes of the laser-EMAT for different metallic materials. After the surface constraint mechanism was adopted, the signal-to-noise ratio of the longitudinal wave increased by at least 13.0 dB, the duration of the main wave was shortened by at least 29.4%, and the amplitude of the main wave decreased by more than 80.5%.

In this paper, in order to improve the defect detection sensitivity in aluminum alloy, an optimized EMAT was designed. An FE simulation model of the electromagnetic ultrasonic excitation acoustic field was established. We studied the influence of important parameters of EMAT on the ultrasonic acoustic field theoretically and obtained the optimized parameters of the EMAT. Artificial defect test blocks were fabricated, and sensitivity test experiments were carried out using the EMAT.

## 2. Equipment and Experimental Materials

### 2.1. Equipment

**Figure 1:**
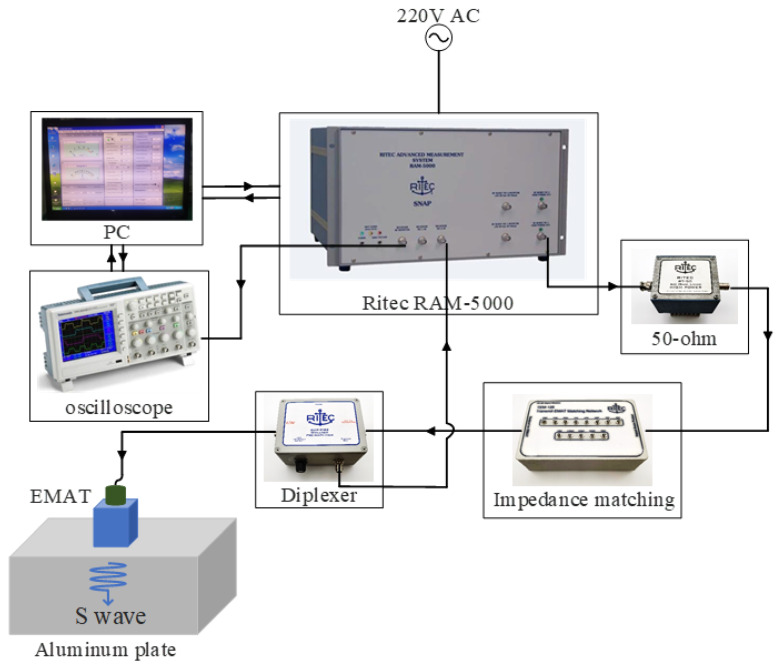
Block diagram of the experimental setup.

The experimental system is shown in Figure 1. It consists of the RITEC RAM-5000 SNAP ultrasonic excitation–reception system, the impedance-matching network, the duplexer, the electromagnetic ultrasonic probe, the test specimen, the oscilloscope, and the computer. The RITEC RAM-5000 SNAP is a high-performance nonlinear high-energy ultrasonic testing system developed by Ritec Corporation (Simi Valley, CA, USA). The RAM-5000 SNAP system excites the probe and receives the ultrasonic signal through the duplexer. The ultrasonic signal obtained by the RAM-5000 SNAP system can be output to the oscilloscope through the built-in signal selector. The oscilloscope transmits the ultrasonic signal to the computer via the USB interface, and the computer acquires the ultrasonic signal through software.

### 2.2. Sample with Artificial Defects

**Figure 2:**
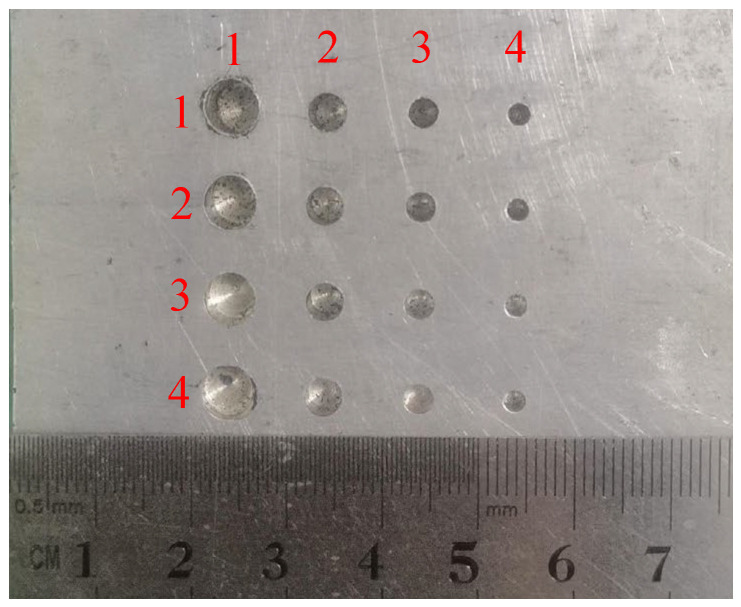
Artificial defect specimen.

This paper focuses on the NDT of non-ferromagnetic materials and optimizes the testing performance of the EMAT. An aluminum alloy plate (40 mm in thickness) was selected as the test specimen. It is 200 mm in length, 80 mm in width, and 40 mm in thickness. The S-wave velocity of this specimen is 3230 m/s, and the L-wave velocity is 6300 m/s. Drills with different diameters were used to drill holes on the same side of the aluminum alloy test block through mechanical processing, as shown in Figure 2. For the convenience of subsequent explanations, we mark the position of each flat-bottomed hole in the order of the horizontal and vertical directions of the flat-bottomed hole array, and present them in the form of coordinates in Table 1. The dimensions of the artificial defects are presented in Table 1.

## 3. Principle of the Butterfly-Coil EMAT

**Figure 3:**
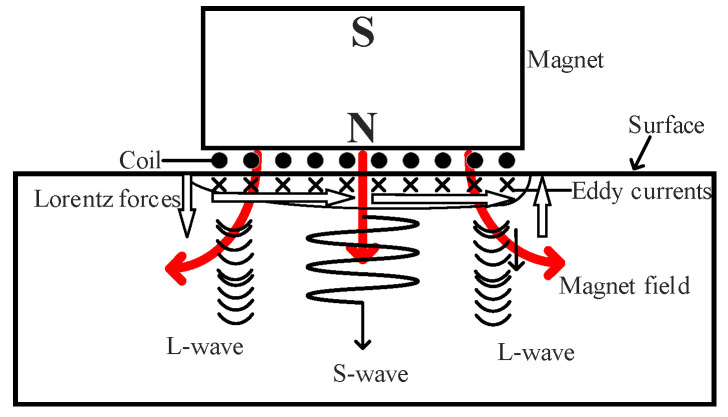
Principle of the butterfly-coil EMAT.

**Figure 4:**
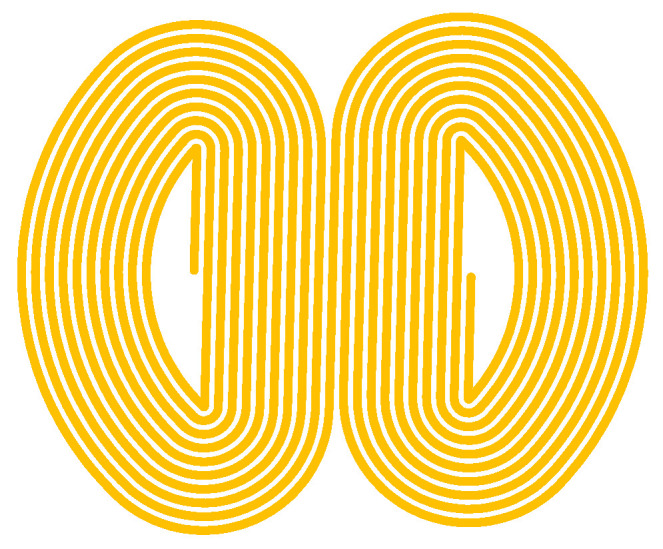
Schematic of the butterfly coil.

In electromagnetic ultrasonic testing, butterfly-coil EMATs are commonly used for defect detection in aluminum alloy plates. The butterfly-coil EMAT consists of a permanent magnet, a butterfly coil, and the surface of the test specimen. In the aluminum specimen, only the Lorentz force acts. The principle of ultrasonic wave excitation by the Lorentz force is shown in Figure 3, and the structure of the butterfly coil is shown in Figure 4.

When a high-frequency alternating current ***J_c_*** is applied to the coil, according to Ampere’s law, an alternating magnetic field will be generated around the coil. At the same time, an alternating eddy current ***J_e_*** is induced in the skin-depth layer of the aluminum block. The Lorentz force acting on the eddy current under the combined action of the bias magnetic field ***B_s_*** of the permanent magnet and the alternating magnetic field ***B_d_*** generated by the coil is as follows:

(1) $$f_{L}=J_{e}\times \left(B_{s}+B_{d}\right)$$

According to elastodynamics, the Lorentz force causes elastic deformation in the skin-depth layer of the aluminum specimen, triggering periodic vibrations of the mass points. The vibration propagates in the form of waves, forming ultrasonic waves. The receiving process is the reverse of the transmitting process. The equation describes the propagation of waves in an isotropic elastic medium with background motion. The inertial force component consists of the elastic restoring force components (driving shear waves and longitudinal waves), convective coupling, and body force. The displacement of the mass points can be obtained by

(2) $$\rho \frac{\partial^{2}u}{\partial t^{2}}=(\lambda_{m}+2\mu_{m})\nabla (\nabla \cdot u)-\mu_{m}\nabla \times \nabla \times u+f_{L}$$

where u is the displacement; $\lambda_{m}$ and $\mu_{m}$ are the Lamé constants; ρ is the density of the tested aluminum alloy specimen.

The operational principle of the butterfly coil primarily relies on its central straight-wire region, where Lorentz forces generate shear waves with vertical polarization. To address electromagnetic interference from the lateral coil segments, a thin copper foil is inserted between the coil and permanent magnet assembly. This eddy-current shielding layer effectively suppresses unwanted current induction in the side-coil areas, minimizing their contribution to the acoustic field. The optimized butterfly-coil design exhibits highly focused energy distribution, with the peak intensity directly beneath the coil center. This configuration preferentially generates S-waves with single-directional particle vibration that is oriented perpendicular to the wave propagation direction and is ideal for defect detection in aluminum alloy plates. A comparative analysis with helical coils highlights the advantages of the butterfly configuration. Although both coil types can excite shear waves, the acoustic field of the helical coil is hollow and conical. At the central position of the helical-coil EMAT, the forces generated by the centrally symmetric coils cancel each other out, creating a region of minimal acoustic field intensity at the center. In contrast, the effective area of the butterfly-coil EMAT is the central parallel wire region. In this central parallel wire region, the current passing through each wire is in the same direction, and the Lorentz forces are superimposed on each other to form a region of maximal acoustic field intensity at the center. This unidirectional focusing characteristic can improve the accuracy of defect detection. However, the butterfly-coil configuration also introduces challenges: the excitation process generates both shear and longitudinal waves simultaneously, with the latter causing detrimental interference in defect signal interpretation. To resolve this issue, a systematic optimization strategy combining finite element simulation and experimental validation was implemented. The key objectives were to maximize S-wave generation efficiency while minimizing longitudinal wave (L-wave) contamination.

## 4. FE Analysis of EMAT

### 4.1. FE Model

In order to evaluate the acoustic field distribution of EMAT, FE analysis was employed. FE is a versatile and powerful tool that allows the investigation of a large number of EMATs with different parameter combinations that would be very costly to fully examine experimentally.

The full two-dimensional (2D) FE simulations were carried out with the COMSOL Multiphysics 6.1 software package. The process of establishing a finite element model can be divided into the following three stages:

(1) Establishing the geometric model: Absorbing boundary conditions were applied to the left and right interfaces of the aluminum plate to ignore the reflection of the structural boundaries. The characteristics of the aluminum plate itself were loaded through the material module. Considering the influence of mesh generation on calculation accuracy, the mesh of the air region was coarsened, and the skin-effect region of the aluminum plate was meshed with more than three layers to meet the grid requirements for analyzing high-frequency electromagnetic fields. To reduce the amount of computation, the coil region was modeled using a uniform multi-turn coil model.
(2) Establishing the electromagnetic field model: The magnetic vector potential field in space was solved for the overall model through Ampere’s law, and the solution domain included the entire space. The remanence model was adopted for the B-H constitutive equation of the permanent magnet.
(3) Establishing the sound-field model: In the solid mechanics module, the Lorentz force was added through the formula to achieve the transformation from the magnetic field to the force field. Appropriate step-size and relative error for transient solutions were selected to ensure the correctness and stability of the solution calculation. The working process of the transducer was the energy conversion from the electric field to the magnetic field and then to the force field realized by the EMAT.

**Figure 5:**
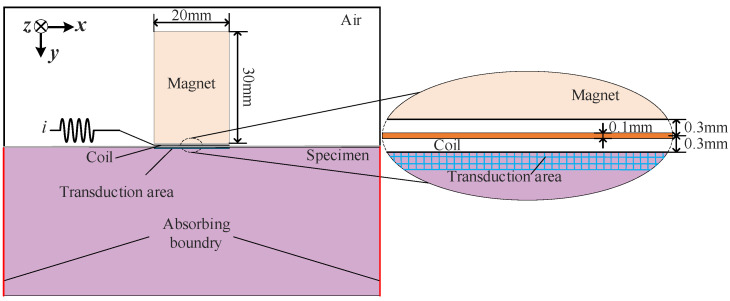
Schematic diagram of EMAT model configuration.

The size of the specimen was 100 mm × 40 mm. The size of the N52 neodymium–iron–boron permanent magnet was 20 mm × 30 mm. The size of the copper coil was 20 × 0.1 mm. The diameter of the wire was 0.1 mm. The distance between the coil and the surface of the specimen was 0.3 mm, which is the packaging thickness of the coil. The distance between the permanent magnet and the coil was 0.3 mm. The frequency of the exciting current *i* was 2.5 MHz. The simulation model of the electromagnetic ultrasonic is shown in Figure 5.

### 4.2. Simulation of EMAT Soundfield

**Figure 6:**
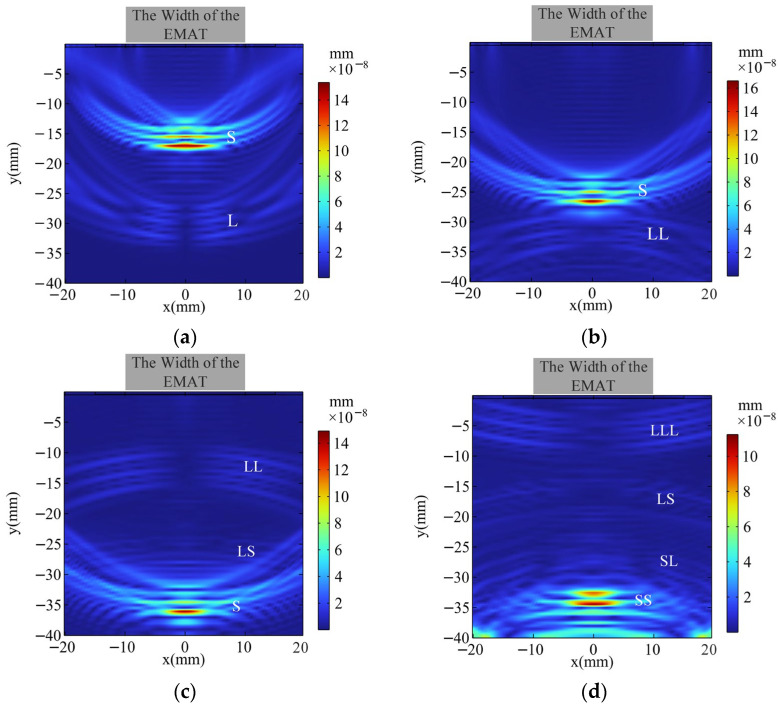
Sound field distribution of the 2D FE simulation model of EMAT at different times: (**a**) t = 6 μs; (**b**) t = 9 μs; (**c**) t = 12 μs; (**d**) t = 15 μs.

The current source is triggered with a 0.5 μs delay and generates a sinusoidal pulse with an amplitude of 20 A and 3 cycles. Through simulation, the propagation process of ultrasonic waves in the aluminum alloy specimen is obtained. Longitudinal waves propagate through the compression and tension of the medium, while transverse waves propagate through shear deformation. The velocity of the longitudinal waves is dominated by the elastic modulus, and the velocity of the transverse waves is dominated by the shear modulus. Since the elastic modulus is usually larger, the velocity of the longitudinal waves is faster. Figure 6a shows the sound-field nephogram at t = 6 μs, where the butterfly-coil EMAT has excited S-waves (S) and L-waves (L) in the aluminum alloy specimen. Figure 6b shows the sound-field nephogram at t = 9 μs, where the L is reflected from the bottom surface of the aluminum block, generating a reflected longitudinal wave (LL) and a longitudinal-to-shear conversion wave (LS). Figure 6c shows the sound-field nephogram at t = 12 μs, where the S is reflected from the bottom surface and undergoes mode conversion, generating a shear-to-longitudinal conversion wave (SL) and a reflected shear wave (SS). Figure 6d shows the sound-field nephogram at t = 15 μs, where the reflected LL is reflected from the top of the specimen, forming a secondary-reflected longitudinal wave (LLL) that propagates back towards the bottom of the specimen.

### 4.3. Simulation of EMAT Signals and Verification

**Figure 7:**
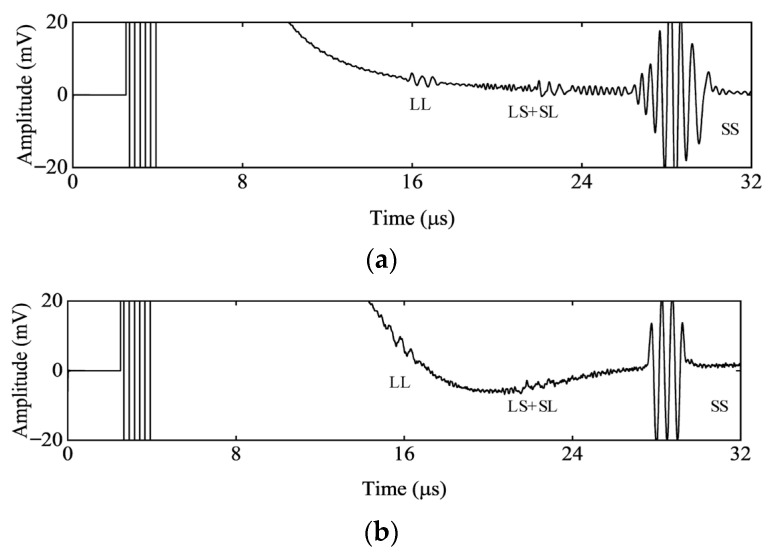
Echo signals from the bottom surface of the aluminum alloy specimen: (**a**) FE simulation signals; (**b**) measured signals.

To quantitatively analyze the sound-field distribution of the EMAT in the FE simulation and verify the effectiveness of the model, the FE model was validated through measurements. The experimental conditions for validation were consistent with those of the FE model. An enamel-coated copper wire with a wire diameter of 0.1 mm (excluding the insulating paint layer). A rectangular N52 neodymium-iron-boron permanent magnet with dimensions of 20 mm × 20 mm × 30 mm is used to provide the bias magnetic field. A sinusoidal pulse output with a frequency of 2.5 MHz, 3 cycles, and a nominal peak-to-peak value of 1400 V (when the output impedance is 50 Ω and the load impedance is 50 Ω) was used. The transmitting impedance matching was adjusted to the optimal condition. Meanwhile, the lift-off distances between the permanent magnet and the coil and between the coil and the specimen were controlled to be the same as those in the model. The FE simulation signals and the measured signals are shown in the Figure 7.

The A signal obtained from the FE model shows a good match with the measured one. Baseline drift is caused by the inertia of strong vibration residues from the main impact of the sinusoidal pulse excitation signal, which cannot be quickly absorbed by the system damping and is random. Since the baseline drift signal is a low-frequency component, it does not affect the amplitudes of the longitudinal wave component and the converted wave component. In the echo signals, each component demonstrates good consistency in both the time domain and amplitude. This indicates the effectiveness of the model design and reflects the distribution law of the sound field.

## 5. Optimization of the EMAT Butterfly Coil

### 5.1. Design of Simulating Experiments

The main purpose of parameter optimization of the EMAT in this paper is to improve the flaw-detection ability of the EMAT. The L-wave interference needs to be reduced, which is quantitatively described by the amplitude ratio of S-wave to L-wave (S/L). In order to obtain the optimal combination of transducer parameters, this paper extracts the key parameters affecting the performance based on the established FE model, analyzes the influence of each parameter on the excitation performance, and conducts an optimization design for the EMAT. Since the FE model has a large amount of calculation and there are many types of EMAT parameter combinations, this paper adopts the orthogonal experimental design method to study the optimization problem of the EMAT. The orthogonal experimental is a design method for studying multiple factors and levels. Based on the principle of orthogonality, test points are scientifically arranged to obtain comprehensive information with fewer test times, revealing the influence rules of various factors on the selected test indicators.

The advantages of the optimization method compared with other optimization methods are mainly reflected in two aspects. Firstly, the orthogonal experimental design is selected as the method to optimize the important parameters. Compared with the comprehensive experiment, it saves a large amount of computational workload and can ensure the reliability of the results. The second aspect is the basis for optimization. Much of the literature focuses only on the amplitude of the echo signal. However, this paper starts from the essential meaning of the transducer and describes the performance of the transducer by the ratio of the amplitude of the transverse and longitudinal wave velocities at the observation points on the main axis sound line.

When the frequency changes, the impedance characteristics of the coil will also change significantly. When the effective width of the coil is fixed, changes in the diameter of the enamel-coated wire used for winding will affect the number of turns of the coil, the total length of the coil, and the cross-sectional area of the coil. Consequently, this will influence the impedance characteristics of the coil, the density of the induced current, and so on. Electromagnetic ultrasound generates ultrasonic signals inside the workpiece through the interaction between the electric field generated by the coil and the magnetic field generated by the magnet. Therefore, the degree of matching between the electric field and the magnetic field will greatly affect the performance of the transducer. The width of the coil is also an important parameter. When other conditions are fixed, changing the effective width of the coil is equivalent to altering the spatial configuration relationship between the electric field and the magnetic field, thus affecting the excitation performance. Based on the above analysis, the factors determined for the orthogonal experiment in this chapter were the main frequency of the excitation signal ***f***, the diameter of the enamel-coated wire ***d***, and the effective width of the coil ***w***. The levels of each parameter are shown in Table 2. The table contains the values of the input factors in the investigated process and corresponding to the four levels adopted.

An orthogonal experiment was designed using the orthogonal table L16 (4^3^). Due to the fact that there are only three factors, L16 (4^3^) can ensure that the main effects are not confounded with second-order interaction effects (such as A × B, A × C, B × C), that is, the resolution is IV. The direction and magnitude of the main effects of each factor can be directly estimated through the column mean differences of the orthogonal array. Based on the common specifications and manufacturing processes of EMAT in aluminum alloy detection, the value ranges of each factor are determined as follows: for f, 2.5~4.0 MHz; for d, 0.03~0.10 mm; and for w, 8.0~20.0 mm. Each of the three factors was taken at four levels, and the orthogonal table was filled. The orthogonal experimental scheme for parameter optimization is shown in Table 3.

### 5.2. Results of Simulating Experiment

**Figure 8:**
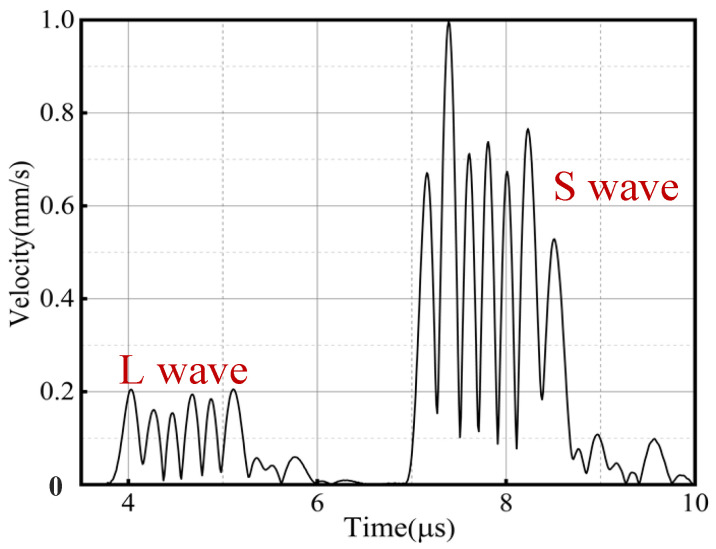
Vibration velocity at the observation point.

Based on the orthogonal experimental scheme and FE model, the optimization process of the EMAT was completed. To save computational time, the simulation of the orthogonal experiment calculated the results within a certain period (3–10 μs) after the ultrasonic pulse was emitted. The intensity level of the transmitted pulse for each parameter was judged by observing the amplitude of the vibration velocity at the center 20.0 mm deep below the aluminum plate, as shown in Figure 8.

By observing the curves, it can be seen that the ultrasonic waves emitted from the EMAT reach the observation point at around 3.5 μs and 7.0 μs, generating vibration wave packets. Based on the sound velocity in the aluminum material, we can calculate that the component reaching the observation point at 3.5 μs is the longitudinal-wave component, while the component reaching the observation point at 7.0 μs is the transverse-wave component. The maximum values of each vibration velocity curve within the two intervals of 3.5–5.5 μs and 7.0–9.0 μs were, respectively, taken. These values were used as parameters to evaluate the vibration intensity and are summarized in Table 4.

In orthogonal experiments, calculating the mean and total values of each factor level is based on the orthogonality and balanced dispersion of the orthogonal array, where each level appears an equal number of times, and the effects of other factors are uniformly distributed and cancel each other out at this level. Through accumulation (total) and averaging (mean), the independent influence of each factor level on the index can be separated, which is used in range analysis to preliminarily determine the significance of the factors. A range analysis was performed on the S/L index in the orthogonal test results, and Table 5 was obtained. In Table 5, ***K*** represents the sum of the test indices corresponding to a certain level of a certain factor, and $\bar{K}$ is the average value of ***K*** with respect to the number of repetitions; ***R*** is the range of a certain factor, that is, $\max\left\{\bar{K}\right\}-\min\left\{\bar{K}\right\}$.

### 5.3. Analysis of Simulating Experiment Results

According to the range analysis, the order of influence of the three factors on the S/L index from primary to secondary is C > B >A. That is, the effective coil width has the greatest impact on the S/L, followed by the coil wire diameter, and the excitation frequency has the least impact. Based on this single index, the optimal combination can be determined as C1B4A4, which means a coil effective width of 8.0 mm, a wire diameter of 0.1 mm, and an excitation main frequency of 4.0 MHz.

A 2D FE analysis model of the butterfly-coil EMAT was established through the COMSOL Multiphysics FE analysis software, and the accuracy and reliability of the model were verified. To ensure the detection sensitivity of the transducer, an EMAT that can excite high-purity transverse waves needs to be designed, and the influence of L-wave should be weakened. Based on this model, an orthogonal experiment was designed to analyze the key parameters that affect the S/L of the transducer through orthogonal experimental analysis, and the degree of influence of each key parameter on the performance was obtained.

### 5.4. Artificial Defects Testing

**Figure 9:**
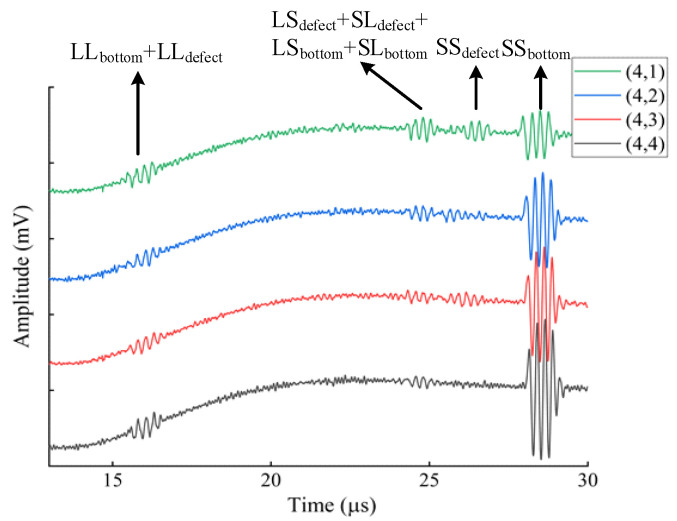
Measured results of the defect array.

The flat-bottomed hole defect array in Figure 2 was measured. There was a total of sixteen flat-bottomed holes. We used the optimized EMAT butterfly coil for the measurement. The size of the permanent magnet of the EMAT was 20 mm × 20 mm × 30 mm, and the parameters of the butterfly coil were manufactured according to the optimization results of the orthogonal experiment: the excitation frequency was 4.0 MHz, the wire diameter was 0.1 mm, and the effective coil width was 8.0 mm. The following figure shows the measurement results of the flat-bottomed holes at the depth of 37.0 mm, with their coordinates being (4,1), (4,2), (4,3), (4,4), and the diameters being 5.0 mm, 4.0 mm, 3.0 mm, and 2.0 mm in sequence. The received signals are shown in Figure 9.

As shown in Figure 9, the analysis of the echo signal components is as follows. At t = 15–17 μs, the signal components are weak longitudinal-wave (L-wave) components, namely, the bottom-surface longitudinal-wave echo (LL_bottom_) and the longitudinal-wave echo of the defect (LL_defect_). At t = 23–25 μs, the signal components that appear are the longitudinal-to-transverse conversion wave of the defect (LS_defect_), the transverse-to-longitudinal conversion wave of the defect (SL_defect_), as well as the longitudinal-to-transverse conversion wave of the bottom surface (LS_bottom_) and the transverse-to-longitudinal conversion wave of the bottom surface (SL_bottom_). As the size of the flat-bottomed hole defect continuously decreases, the amplitude of its echo signal weakens continuously. At t = 25–27 μs, the signal component is the shear wave echo of the defect (SS_defect_). As the diameter of the flat-bottomed hole decreases, the reflection area of the defect reduces, so the energy of the reflected wave weakens. Moreover, the EMAT can detect a flat-bottomed hole defect at a depth of 37 mm from the surface and with a diameter of 3 mm, and the SNRs of 1, 2, 3, and 4 are 12.41, 8.42, 8.13, and 4.32, respectively. Within this depth range, the echo of the flat-bottomed hole defect with a diameter of 2 mm is almost invisible, and the SNR is extremely low. At t = 27–29 μs, the signal component is the bottom-surface transverse-wave echo (SS_bottom_).

Under the experimental conditions of this paper, the limit sensitivity that the structurally optimized transducer can detect is a flat-bottomed hole with a depth of 37.0 mm and a diameter of 3.0 mm.

## 6. Conclusions

An FE simulation model was established for EMAT testing. Using the model, the visualization EMAT acoustic field can be obtained, and the tested signal can be predicted. There was good correlation between the simulated signals and the measured signals. A parameter optimization plan for the butterfly coil was designed, and the optimal parameters were obtained through FE simulation. An EMAT was fabricated using the optimal parameters obtained from theoretical research, and a specimen with artificial defects was tested. The EMAT is capable of detecting a flat-bottomed hole with a diameter of 3 mm at a buried depth of 37 mm in the aluminum specimen. This paper optimizes the EMAT and improves the detection performance of the EMAT for internal defects in aluminum alloys by increasing the amplitude ratio of transverse and longitudinal waves.

Regarding subsequent research directions, we have the following prospects and considerations: Conduct research on the sensitivity and accuracy of detecting artificial defects of different types and sizes, and further expand the scope of detection of objects to naturally occurring defects in aluminum alloy plates. Develop a high-temperature online detection system. Aiming at the real-time detection requirements during the rolling process, develop high-temperature-resistant EMAT sensors and an integrated detection platform to achieve dynamic defect monitoring on the production line.

## Figures and Tables

**Table 1:** Dimensions of artificial defects in experimental specimens.

Coordinate	Diameter (mm)	Machining Depth (mm)
1,1	5.0	9.0
1,2	4.0	8.9
1,3	3.0	9.1
1,4	2.0	8.8
2,1	5.0	7.1
2,2	4.0	6.8
2,3	3.0	7.0
2,4	2.0	7.0
3,1	5.0	5.1
3,2	4.0	5.1
3,3	3.0	5.0
3,4	2.0	4.9
4,1	5.0	3.0
4,2	4.0	3.1
4,3	3.0	3.2
4,4	2.0	2.9

**Table 2:** Factor levels of the orthogonal test for EMAT optimization.

Level	f (MHz)	d (mm)	*w* (mm)
1	2.5	0.03	8.0
2	3.0	0.05	12.0
3	3.5	0.08	16.0
4	4.0	0.10	20.0

**Table 3:** Orthogonal test scheme for EMAT parameter optimization.

Test No.	*f* (MHz)	*d* (mm)	*w* (mm)
1	2.5	0.03	8.0
2	2.5	0.05	12.0
3	2.5	0.08	16.0
4	2.5	0.10	20.0
5	3.0	0.03	12.0
6	3.0	0.05	8.0
7	3.0	0.08	20.0
8	3.0	0.10	16.0
9	3.5	0.03	16.0
10	3.5	0.05	20.0
11	3.5	0.08	8.0
12	3.5	0.10	12.0
13	4.0	0.03	20.0
14	4.0	0.05	16.0
15	4.0	0.08	12.0
16	4.0	0.10	8.0

**Table 4:** Results of orthogonal experimental simulation.

Test No.	L (mm/s)	S (mm/s)	S/L
1	1.910	14.896	7.7977
2	2.327	17.192	7.3865
3	2.296	20.682	9.0067
4	3.628	15.433	4.2536
5	0.933	7.083	7.5847
6	2.067	17.115	8.2801
7	1.951	8.764	4.4912
8	2.468	21.362	8.6531
9	0.565	3.987	7.0557
10	0.841	3.683	4.3793
11	1.640	13.651	8.3233
12	1.120	10.529	9.3975
13	0.495	1.713	3.4600
14	0.828	5.065	6.1163
15	0.563	8.033	14.2480
16	1.057	16.056	15.1832

**Table 5:** Range analysis of the simulation results of the orthogonal experiment.

	A (*f*)	B (*d*)	C (*w*)
***K***	28.444	25.898	39.584
	29.009	35.560	39.028
	29.156	36.069	30.832
	39.007	37.487	16.584
$\bar{K}$	7.111	6.475	9.896
	7.252	8.890	9.757
	7.289	9.017	7.708
	9.752	9.372	4.146
***R***	2.641	2.897	5.750

## Data Availability

The original contributions presented in this study are included in the article. Further inquiries can be directed to the corresponding author.
